# Wildfires as legacies of agropastoral abandonment: Gendered litter raking and managed burning as historic fire prevention practices in the Monte Pisano of Italy

**DOI:** 10.1007/s13280-024-01993-x

**Published:** 2024-03-08

**Authors:** Andrew S. Mathews, Fabio Malfatti

**Affiliations:** 1 Department of Anthropology, Room 325, Social Sciences Building 1, University of California, Santa Cruz, CA 95064 USA; 2 Centro Ricerche EtnoAntropologiche, Via Della Maulina 826, M.S. Quirico, 55100 Lucca, Italy

**Keywords:** Agropastoral management, Gender, Litter raking, Peasants, Traditional ecological knowledge, Wildfires

## Abstract

Agropastoral practices that historically reduced the flammability of Mediterranean landscapes are poorly understood due to state prohibitions and lack of scientific interest. Oral histories, analysis of agronomical writings, transect walks, and ethnographic study of fire managers and community members in the Monte Pisano of Italy, find legacies of traditional agropastoral practices in present-day landscapes. Forest leaf litter raking, largely carried out by women, combined with fire wood cutting and burning to greatly reduce fire risk. Historic stigmatization of traditional burning and ignoring gendered peasant labor have reduced contemporary scientists’ and fire managers’ understandings of ecological processes and of options for reducing fire risk. Fire managers in the Mediterranean, and in areas around the world affected by rural depopulation, would benefit from a better understanding of traditional agropastoral and fire management practices. Litter raking has been understudied outside Central Europe, is often gendered, and may have important ecological consequences around the world.

## Introduction

In 2023, as in recent years, there has been an epidemic of large forest fires across the Mediterranean. Forest fire area has been increasing in recent years (European Commission Joint Research Centre et al. 2022). It is likely that heat and aridity caused by climate change will make fires larger, hotter, and more dangerous in the future (Michetti and Pinar 2019; Grünig et al. 2023). A major contributor to contemporary fires is the abandonment of traditional smallholder agriculture and pastoralism, which has caused the colonization of grasslands by scrub and trees, and the replacement of repeatedly burned forest by flammable scrub (Valese et al. 2014; Ferrara et al. 2021). The Mediterranean landscape has long been shaped by complex agrosilvopastoral systems (Blondel 2006, 2010). The abandonment of these practices since the 1960s has produced increasingly flammable landscapes. Detailed ethnohistorical research with shepherds and peasants reveals practices that formerly produced less fire-prone landscapes. Through oral history of former practices of leaf litter raking (mainly by women) and of managed burning, in the Monte Pisano of Central Italy, we show how material legacies of former land use practices, combined with cultural legacies of state prohibitions, have set the stage for current large wildfires and community responses. We argue that a better understanding of former land care practices might suggest ways of making landscapes less fire prone in the future. Our limited understanding of these practices is a cultural legacy of the lack of state interest in peasant practices, especially by women, and of the stigmatization of traditional agricultural and pastoral burning.

## Theoretical framework

Anthropologists and political ecologists have long been concerned with conflicts over control of natural resources (Peluso and Vandergeest 2015) and with epistemic differences between rural people, scientists, and officials about fire use in agricultural systems (Dove 1983; Fairhead and Leach 2000; Mathews 2011). States around the world have been hostile to traditional managed burning^1^ practices by peasants and indigenous people (Pyne 1997). This hostility has not necessarily prevented burning, but it has created scientific and official ignorance as how, when, and where people burned (Mathews 2005). Traditional managed burning by shepherds and rural people across the Mediterranean has been illegal, stigmatized, and little studied, although case studies from the French Pyrenees, Liguria, and Spain suggest that it was formerly widespread (Seijo et al. 2015; Cevasco et al. 2020b; Metailie et al. 2020). Even where rural agricultural practices are not actively forbidden, they can escape official and scholarly attention if they are considered backward or nonmodern as in the case of swidden agriculture in South East Asia (Dove 1985). In Italy fascist projects of drainage and wheat production led to lack of attention to traditional forms of agriculture (Armiero et al. 2022), and managed burning has been strongly stigmatized (Ascoli and Bovio 2013). With the rapid industrialization of agriculture after the war, traditional smallholder and pastoral practices, including controlled burning, were ignored. When agricultural practices are carried out by women and children, a further marginalization can take place, as gendered labor and property relations can be ignored by legal systems and officials (Carpenter 1991; Carney 1996; Rocheleau and Edmunds 1997). This is what appears to have happened with litter raking in the Monte Pisano of Italy. Our study redresses the official marginalization of traditional land use practices in general, and particularly, of practices carried out by women.

The interdisciplinary field of historical ecology has long been concerned with studying landscape change through an eclectic use of methods from the social and natural sciences (Crumley 2017). Chosen methods can vary depending upon the composition of the research team, time depth, and available data, but the landscape is often the unit of analysis. Historical ecologists, like anthropologists, have been concerned with the role of human cognition and action in shaping landscapes (Cevasco and Moreno 2015; Moreno 2018) with the biographies of landscapes, and with changing social memory. A key challenge of historical ecology is to combine different kinds of evidence and to recognize the strengths and limitations of each, without forcing too much agreement between them (Crumley et al. 2017). In our case study, oral history, census data, and a comparison with quantitative evidence from the Swiss Alps (Gimmi et al. 2008) allow us to extrapolate the quantitative impacts litter raking and grazing upon forests. Historical ecologists of the Mediterranean are certain that there is a long history of fire use, as recorded in soil charcoal (Valese et al. 2014), but there is less information about practical reasons for burning. Our study partially addresses this gap.

## Materials and methods

We combine a review of agricultural history and nineteenth- and twentieth-century agronomical writings, oral histories with elderly rural people in the Monte Pisano of Central Italy, ethnographic interviews with residents and firefighters, and transect walks that provide qualitative descriptions of contemporary forest conditions. Between 2016 and 2019 we carried out interviews with ten men and seven women, born between 1928 and 1956 in communities around the Monte Pisano, focusing on former agricultural and pastoral activities, including firewood gathering, charcoal burning, fruit and herb gathering, pine resin gathering, and chestnut cultivation. We carried out ten interviews with government officials, local residents, fire managers, and government officials in the wake of large wildfires in 2018, as well as long-term participant observation in community events from 2018 to the present.

### The study region: The Monte Pisano

**Fig. 1:**
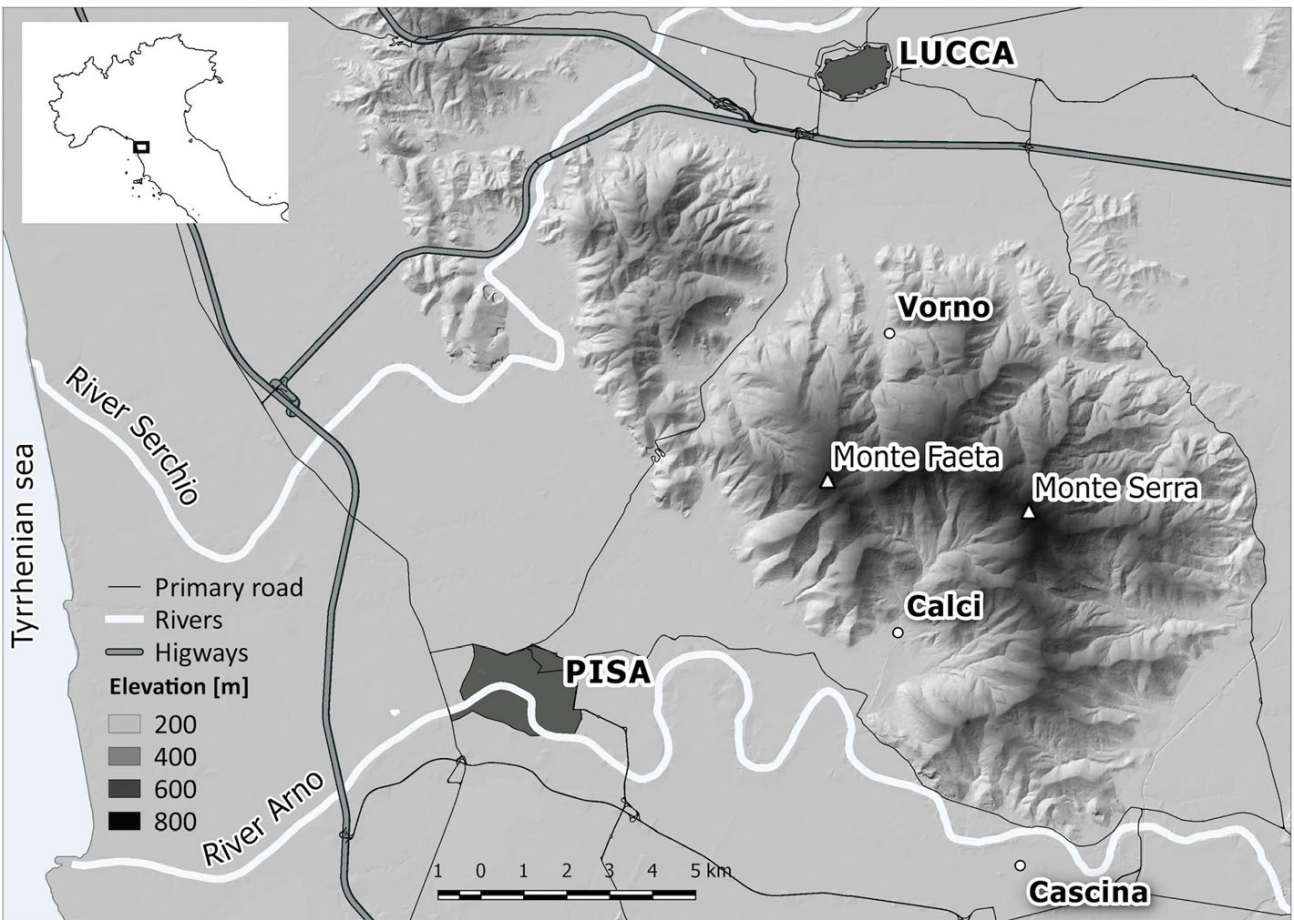
Location of the Monte Pisano

The Monte Pisano is a modest sized mountain range of about 15 000 hectares, located between the cities of Pisa and Lucca in Central Italy (see Fig. 1). These mountains are outliers of the main chain of the Apennine mountain range. Elevation ranges from 15 to 917 m above sea level. Climate varies with elevation and is in the Köppen Csa, class, humid temperate with dry summers, Mediterranean subtype. Average temperatures range from 3C in coldest month range to over 22C for the hottest. Precipitation varies from 928 mm in the plain to 1203 mm at the crest of the mountains. Several small towns occupy the valleys. On the lower slopes and on valley floors agriculture predominates, with olive cultivation particularly widespread on the southern side of the mountains. Lower elevation forest is dominated by maritime pine (*Pinus pinaster*) with an understory of gorse (*Ulex europaeus*) and tree heather (*Erica arborea*), with some patches of oak (*Quercus pubescens*). Higher up there are mixtures of chestnut (*Castanea sativa*) and maritime pine with chestnut prevailing above 500 m. Along river galleries more mesic vegetation, including manna ash (*Fraxinus ornus*), and hornbeam (*Ostrya carpinifolia*) dominate. On dryer, south facing slopes, especially at lower elevations, there are areas of macchia scrub, dominated by tree heather and strawberry tree (*Arbutus unedo*) with patches of cork oak (*Quercus suber*) and holm oak (*Quercus ilex*), while on areas of limestone bedrock there is Mediterranean garrigue dominated by *Phillyrea angustifolia, Pistacia lentiscus,* and *Myrtus communis*. Much of the crest of the mountain is covered with exotic conifer plantations (*Pinus nigra, Cedrus libani*). At lower elevations on the northern and eastern slopes there are bands of the invasive black locust (*Robinia pseudoacacia*). For a complete description see (Bertacchi et al. 2004).

## Results and discussion

### Historical evidence of burning and litter raking

Forestry regulations in Italy, as in other European countries, have historically been concerned with timber and firewood production and have been hostile to managed burning (Pyne 1997). In the Monte Pisano state interest in naval timber production caused burning to be forbidden as early as 1571 (Granducato di Toscana 1571). The fascist regime in Italy (1923–1943) further intensified longstanding state hostility to fire through regulations that prohibited burning within forests (Consiglio Provinciale dell' Economia 1931). These restrictions have created official ignorance and limited scientific research on the rationale, location, and types of burning which formerly produced less fire-prone landscapes. Traditional burning was done for different reasons at different places and times: to clear heath and brambles from mountain pastures (Cevasco et al. 2015a, b), to burn leaves in cultivated chestnut groves before harvests (Mathews 2022), in swidden agriculture known as *debbio* (Cevasco 2004), to manage heathlands and larch woodlands in the Alps (Valese et al. 2014), to burn stubble after the harvest on grainlands, and to burn grass on earthen terrace walls. The abandonment of traditional managed burning has contributed to an increase in forest and scrub area and to increased fuel loads within woodlands, making landscapes more fire prone.

Among a myriad traditional agricultural practices, forest leaf litter raking for fertilizer had a particularly important role in reducing the flammability of Apennine forests. Leaf litter raking is well known for Switzerland and the Rhone valley (Gimmi et al. 2008; Bürgi et al. 2013). In Central Europe litter raking was closely regulated by the state, leaving a record of conflicts over a valued resource in Moravia (Szabò 2021). Litter raking and wood pasture have left material legacies in fertility, growth rates, and carbon absorption of present-day forests (Gimmi et al. 2013). Litter raking is also recorded in nineteenth-century agronomical writings in Italy as a minor practice (Mazzarosa 1846; Bertagnolli 1889). In early modern Italy tree leaves had many uses, from shredding for animal fodder (Cevasco 2004), to litter raking for fertilizer, and forests were usually grazed by animals (Moreno 1990, 2018; Grove and Rackham 2001). Litter raking is recorded until after World War II in the foothills of the Italian Alps and the Cerbaie near the Monte Pisano (Piussi and Stiavelli 1986; Volta 1989). Until the 1960s chestnut leaves were raked for stable bedding in Liguria and the Apennines (Moreno 1990; Mathews, 2022) and pine needles were cut for fertilizer in Liguria (Pescini et al. 2018). Whereas in Switzerland litter raking increased in the late eighteenth century and peaked in the 1930s, with a rapid decline after World War II (Gimmi et al. 2008), litter raking in Italy may have a much longer duration, as demonstrated by sixteenth-century communal litter raking regulations in Liguria (Raggio 1992, 1995). In the many areas of Italy where densely terraced landscapes coexisted with large numbers of sheep, nearby forests were a source of fertilizer as well as of pasture, kindling, firewood, charcoal, and construction timber. In grain-producing areas abundant straw made litter raking less necessary. Critically, in Mediterranean summer dry climates, litter raking produced forest landscapes that were open, clear of woody debris, and much less likely to burn.

In spite of solid evidence of widespread litter raking across the Northern Apennines and the foothills of the Alps, there is relatively little present-day scientific and official knowledge of this practice, nor of its legacy effects upon landscape flammability. This cultural legacy of nonknowledge is due to the combined effects of scientific interest in trees for timber and firewood production, to the fact that litter raking was a nonmarket practice that paid no taxes, and to the effects of state ideologies of agricultural modernization according to which peasant practices were marginal and doomed to disappear. Forestry regulations from 1931 mention litter raking in passing as a ‘secondary forest product’ (Consiglio Provinciale dell' Economia 1931). Forestry statistics record firewood and timber production but make no mention of leaf litter. An additional reason for its neglect is that litter raking was largely carried out by women and children.

### Oral histories of land care and abandonment

**Fig. 2:**
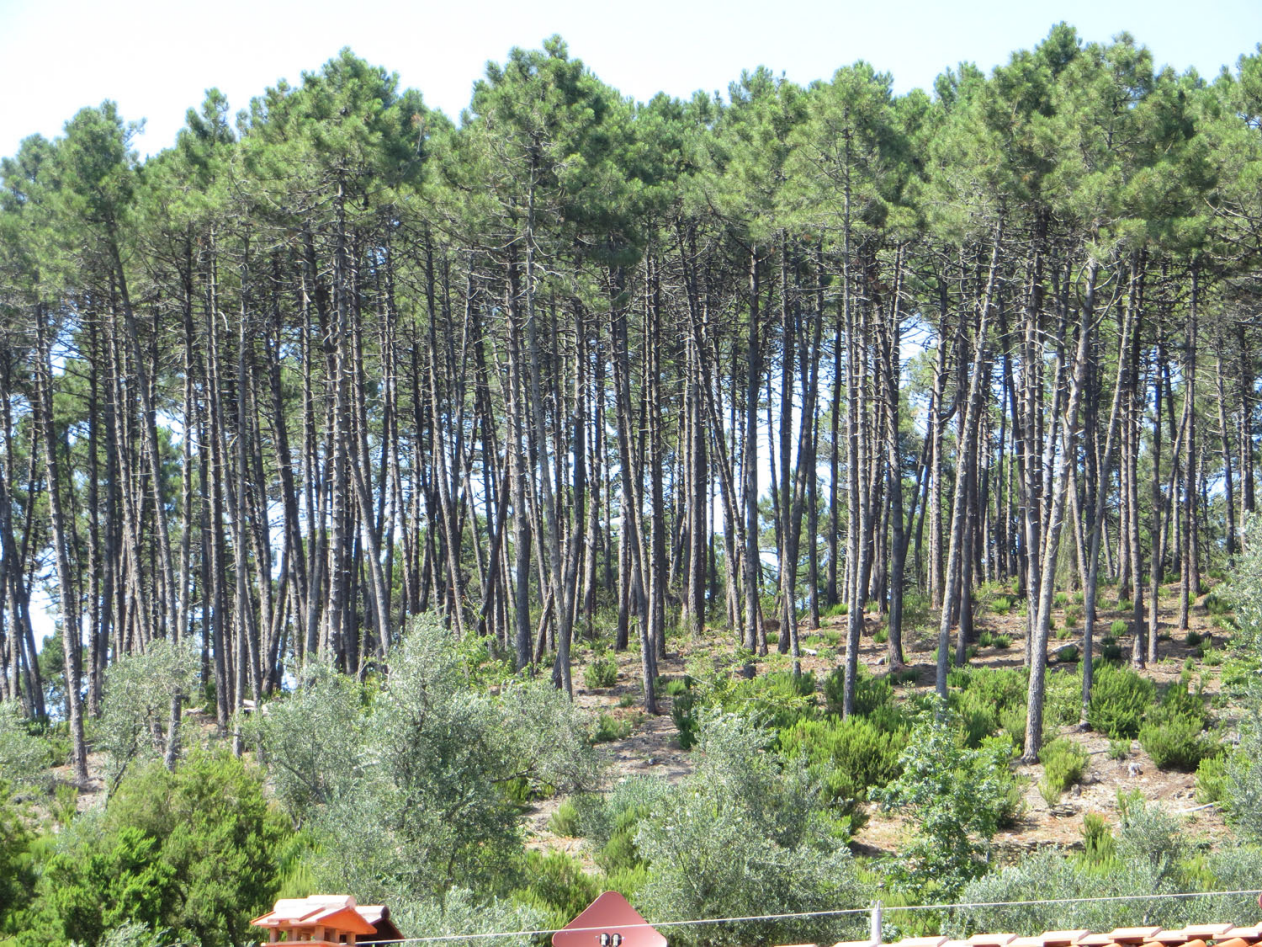
Stand of maritime pine with tree heather beneath. Vorno, Monte Pisano (Picture by author, 2019)

The elderly peasants we talked to described the present-day landscape as abandoned, overgrown, and fire prone. They contrasted this with the landscape that they grew up in, which was busy and full of people, including women and children. People gathered firewood and charcoal, raked leaf litter and cut ferns for stable bedding, cut brush for kindling, gathered herbs, berries, and mushrooms, and rapidly extinguished small wildfires. After the war, maritime pine trees were tapped for resin in some areas. Every scrap of wood or vegetation had some use. Pastures were closely cropped by sheep, and the forest floor was cleared of fallen branches, leaf litter, and shrubs. Forests were grassy and heavily grazed, and in places the soil was bare, with only a light covering of leaves or pine needles. Our informants described the landscapes of the Monte Pisano as having been ‘clean,’ comparing it unfavorably with the ‘dirty’ conditions of the present. As one woman told us ‘you could walk almost everywhere, whether there was a path or not’ and ‘whether because of litter raking, or... if a tree fell someone immediately made it into firewood’ (Lenzarini 2019).^2^ The arrival of fossil fuel energy changed the forest. ‘When heating came in, everything, everything was abandoned, [now] a tree falls and stays in the woods, you can’t pass anymore, and it is harder to put fires out’. Woodland and pasture properties were clearly demarcated by trees and stones and invading others’ land was out of the question. There was little fire. As one woman remarked ‘What fires? What did it burn if there was nothing on the ground? Everything was cleaned’ (Bonanni 2019a). Another woman stated: ‘It didn’t burn before. You could even leave a cigarette, but it was so clean, there was not even a stick, not a blade of grass, not a bush, there was nothing, it was all clean’ (Lenzarini 2019). The heavy presence of people working the land meant that rare small wildfires were rapidly extinguished. Something of what these tended forests might have looked like can be seen in the pine forests in Fig. 2, where understory oak and scrub have been removed to reduce fire risk, creating a more open, parklike appearance.

**Fig. 3:**
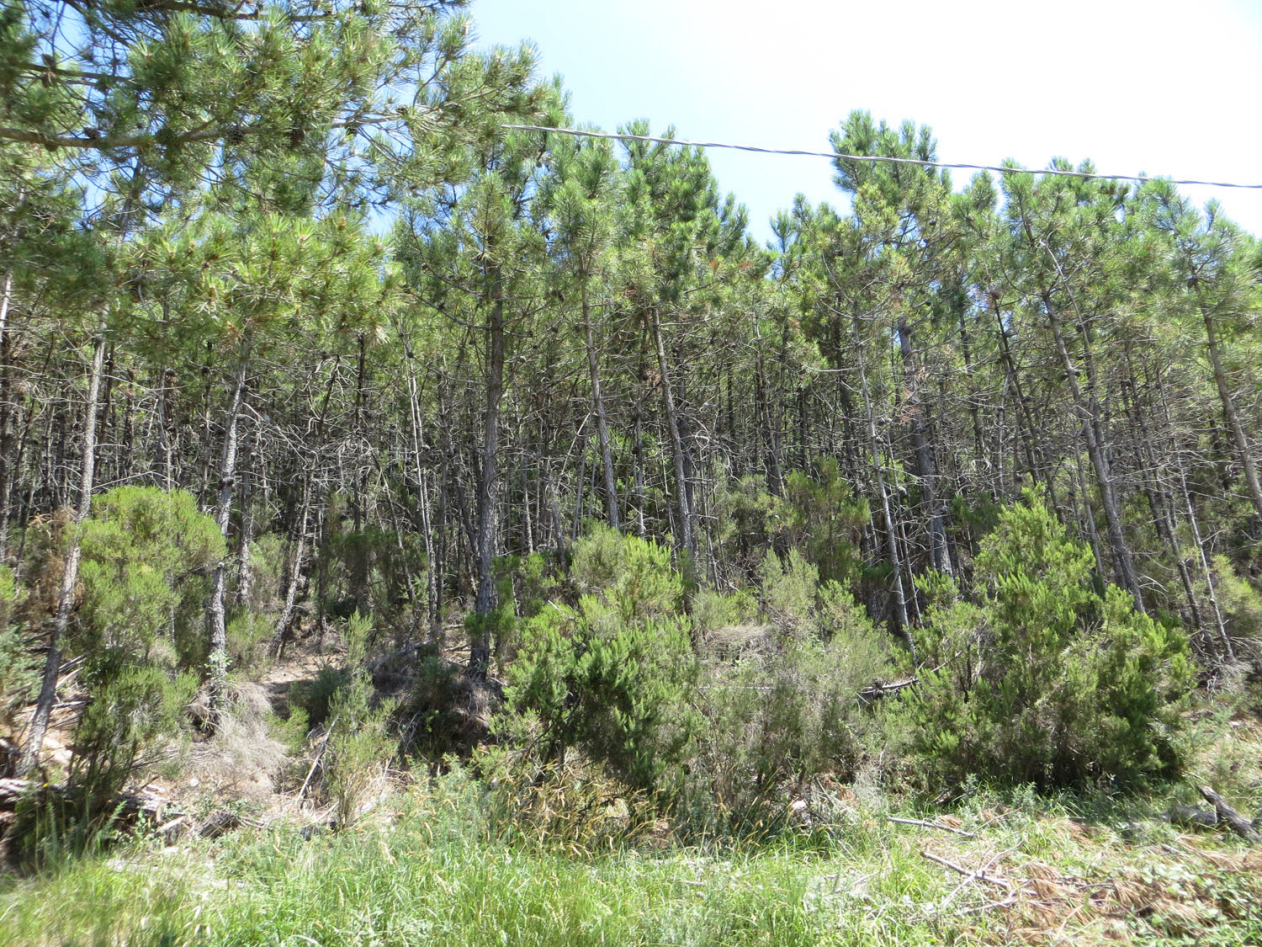
Young maritime pines with an understory of tree heather, Monte Pisano (Picture by author, 2016)

Most present-day forests have a dense layer of understory scrub with thick layers of leaf litter on the forest floor, as seen in Fig. 3.

**Fig. 4:**
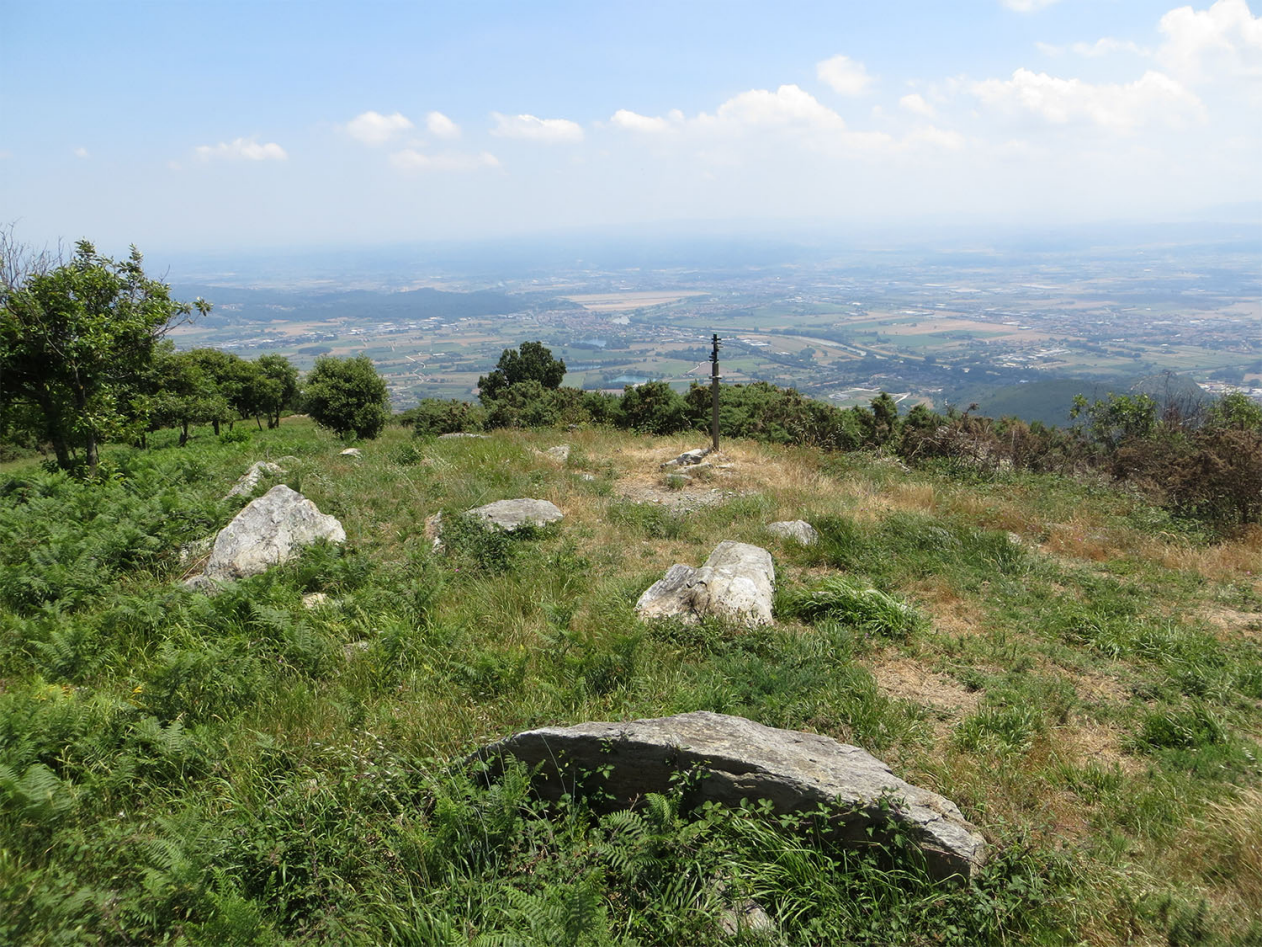
Grassland on the crest of the Monte Pisano after a 2015 prescribed burn (Picture by author, 2016)

The decline in pastoralism allowed the colonization of mountain pastures by scrub and trees, while many pastures have been planted with exotic conifer plantations. A recent prescribed burn shows us what this kind of pasture might have looked like (Fig. 4).

Litter raking linked forests with olive groves through the work of humans who herded sheep and raked litter. Most share cropper households cultivated olive trees on terraces and combined sheep manure and forest leaf litter to produce fertilizer. Each farm household, with perhaps 3 to 5 hectares under cultivation, would keep a small herd of 10–50 sheep who were stabled at night. Every day or two, the stable bedding of leaf litter, mixed with animal dung and urine, was removed. The mixture of litter and manure was composted into fertilizer and dug into the roots of olive trees every other year, producing a deeply anthropogenic soil down to about thirty centimeters. During the day animals grazed pastures on the crest of the mountains, in woods, on olive terraces, along roads, and on open fields on the valley floor, depending upon the season. Litter raking was essential to olive cultivation and households with no animals made arrangements with visiting shepherds. Complex and constantly changing systems of exchange and intermarriage linked shepherds and peasants (Giambastiani 2021). While it was shepherds who paid farmers for grazing permission with cheese in the twentieth century, in the nineteenth century it was farmers who paid shepherds in cash (Mazzarosa 1846, 88). Litter raking to produce fertilizer allowed dense olive cultivation in a hilly region with little grain cultivation, and hence, little straw for stable litter. As Table 1 shows, our informants were nearly unanimous in recalling the former importance of leaf litter raking. The sole exception was a woman who had grown up in a small town and had less experience of rural work. An additional focus group of five women, born between 1939 and 1955, agreed that litter raking had been a universal activity. Litter raking continued until the 1960s, when first young men and then women left farming to take up industrial work in factories, and chemical fertilizers replaced animal compost.

**Table 1:** Litter raking by gender

	Men *N* = 10	Women *N* = 7
Reported litter raking as a common practice	10	6
Raked leaf litter themselves or saw close family members do so	2	6
Did not report litter raking	0	1

A closer reading of our interviews strongly suggests that it was women and children who did most of the litter raking among this generation born between 1928 and 1956. It was almost entirely from the women we talked to that we heard the practical details of litter raking, and of how it fit into the cycles of daily and seasonal work. As Table 1 shows it was mainly women who remembered raking leaf litter themselves, or who gave detailed accounts of close family members doing so. Lucia Massoni, born in 1935, told us how her grandmother gathered litter with a wooden hand rake, and how litter was carried home by her grandfather on a donkey (Massoni 2019). Maria Lenzarini, born in 1928, raked leaf litter every morning before school, beginning when she was about five years old (Lenzarini 2019). Silvia, who was born in 1928, told us how she would rake leaf litter with two or three friends every morning (Bonanni 2019b). Heavy baskets or bundles of leaf litter had to be carried three or four kilometers on most days. Leaf litter consisting of pine needles could be tied up in bundles, but some people recalled using baskets*.* Leaf litter was piled ready for use outside stables, in a long mound resembling a haystack.

The detail of these women’s description is in striking contrast to the vaguer accounts from men. The men, born between 1936 and 1956, described litter raking as something that they knew about, but only two remembered having done it themselves. A man who grew up in a sharecropper family recounted that ‘litter raking was done’, another, born in an estate agent family*,* described it as ‘done by peasants’. Only two described litter raking in more detail. Fausto Giorgi, born in 1950 on a farm well outside the Monte Pisano, told us that the estate agent demanded that woodlands be kept clear by raking and burning, to facilitate water flow and prevent floods and landslides (Giorgi 2019). Fabio Casella, born in Calci in 1956, remembered seeing litter raking as a child.

Litter raking may have been women and children’s work well before the twentieth century. We know that in peasant households across Italy as far back as the eighteenth-century ‘heavy’ work such as plowing and digging was supposed to be done by men, although what counted as ‘heavy’ varied by region and time (Palazzi 1990, 1997, 321–342). In the Monte Pisano for example, the burdensome task of carrying firewood to the city of Pisa in the late nineteenth century was women’s work (Pelosini 1970 [1890]). Our interviews tell us that it was women who harvested olives from the ground, gathered kindling, and cut animal fodder and ferns, and raked leaf litter, all activities which would have been classified as ‘light’ work. In any case, gendered labor practices responded to changing household composition and economic opportunities. When men were away at war, injured, or drawn into industrial employment after World War II, women did ‘heavy’ men’s work too.

This generation of former peasants likely lived with particularly feminized agricultural labor. Although off farm employment was against the terms of share cropping agreements (Biagioli 2002), by the 1950s the lack of tenants was causing landowners to turn a blind eye. In the 1960s in the Monte Pisano, in many sharecropper households men worked in factories during the week, and did farm work on evenings and weekends, leaving women and children responsible for the rest. Combining on and off farm work has been a strategy of land owning peasants across Europe for centuries, often with increased on farm work for women (Holmes 1983). Women in landless households may have moved more quickly into waged labor, from spinning and weaving, to washing clothes for the cities of Lucca and Pisa. Men from landless households could find work in small rural industries, from paint manufacture to olive mills (Massoni 1999), or by migrating to more distant cities. Although the feminization of litter raking may have been a response to relatively recent economic shifts, it is likely that it has been ‘women’s work’ for much longer. Verses recounted by a peasant woman born in 1861 near Firenze describe litter raking as stereotypically a peasant woman’s work.

If I choose to be a peasant woman I will always gather leaf litter, I’ll turn as black as tar, I don’t want to be a peasant woman.

If choose to be firewood cutter I will always be lamenting, I will become a beast of burden, I don't want to be a firewood cutter (Contini 2012)

### Quantitative estimates of litter raking

Qualitative accounts of litter raking can be combined with quantitative estimates of sheep populations to give an estimate of the quantity of biomass that was removed. The Monte Pisano now has about 200 sheep, grazed in abandoned olive groves on the lower slopes and on fallow agricultural fields on the plains. This is a stark contrast with the agricultural census of 1929, which records 8032 sheep for the three communes on the Pisa side of the Monte Pisano (Istituto Centrale di Statistica del Regno D'Italia 1933b). On the Lucca side of the mountains there were 4525 sheep divided between the Monte Pisano and the nearby Pizzorne mountains (Istituto Centrale di Statistica del Regno D'Italia 1933a). A reasonable estimate of the sheep population of the Monte Pisano in the early 1930s is about 10 000 animals with much smaller numbers of goats. These numbers roughly agree with our interview data, which describes almost all farms as having a flock of 10–50 sheep, with additional animals hosted during winter visits by shepherds from the high Apennines. One retired shepherd estimated that there were 1500 sheep on one side of the valley of Calci in the 1950s, which agrees well with the census figure of 3530 sheep for the entire valley. Census numbers are likely to be an underestimate, because peasants and shepherds tried to avoid taxes, because many animals were only present for part of the year, and because of distrust between shepherds and the state. The fascist regime of Benito Mussolini (1923–1943) saw grazing by goats and sheep as destructive of forests and had targeted goats with heavy taxes in the 1927 grazing regulations (Armiero 2011, 129–134). Burning within one hundred meters of woodland was severely fined (Consiglio Provinciale dell' Economia 1931). Mountain pastures were targeted for afforestation with conifer plantations where grazing by sheep and goats was banned. In this atmosphere of conflict and distrust between shepherds and the state, counting grazing animals was extremely difficult. The census reports are not any more reliable than our informants’ statements that every farm household had ‘ten to fifty sheep’. The combination of the two is more convincing than either alone.

Between oral history and census is possible to extrapolate the quantitative impact of litter raking upon forests. For the Pisa side of the mountains for which we have detailed figures, there would have been 2.2 animals/ha across woods and pastures, comparable to the 2.5–3.5 animals/ha recorded for the Valais region of Switzerland in the 1930s, where litter raking and wood pasture were similarly prevalent (Gimmi et al. 2008). Taking the lowest estimate for biomass removal by litter raking in the Valais case (Gimmi et al. 2008) of 0.16 kg/m^2^/year, this would have been 30–40% of the 0.4–0.5 kg/m^2^/year of net primary productivity (Maselli et al. 2009) for fertilizer, with additional biomass extracted by wood pasture. When we add the effects of firewood and brush cutting and the removal of fallen wood, our informants’ statements that the forest floor was almost bare soil are completely credible.

The long-term effects of litter raking on soil fertility and forest productivity have long a concern to Central European and Italian foresters (Piussi and Stiavelli 1986; Szabò 2021). In the Monte Pisano litter raking may have left a legacy of reduced soil carbon storage and forest growth rates, as in the Swiss case described by Gimmi and colleagues (Gimmi et al. 2013), but it may also have increased grass productivity in woodlands (Moreno 1990). In the case of the Monte Pisano, and in the summer dry climates of the Apennines, litter raking also made pine forests less likely to burn. Continued litter raking allowed otherwise highly fire-prone maritime pine to dominate lower slopes where pine had displaced chestnut in the nineteenth century (Casazza et al. 2021; Mathews 2022). A cultural landscape of flammable pine forests was kept relatively fire free as long as litter raking continued and people were present to put out small fires.

### Managed fire and managed grazing

If forests were made less flammable by litter raking, it was a combination of cultivation, grazing, and managed fire which kept olive groves and agricultural fields clear of flammable vegetation. Several older women told us they burned grass on the slopes of terraces where scythes and sickles could not easily reach. Our interviews were carried out immediately after a major wildfire, and even elderly peasants and shepherds very unwilling to talk about managed burning. Instead they blamed fires upon incompetence, criminality, and landscape abandonment.

One exception is the former peasant Maria Lenzarini, talking about burning terraces. ‘When I burn I am safe. For me it does not get away. Maybe I bother people with the smoke. But peasants should support this because they are peasants. And now that there are no peasants left smoke bothers people. Two or three times the foresters came. The saw, they looked, they said “Do me a favor and put it out”. I put it out but they didn’t fine me’ (Lenzarini 2019). Several other older women told us that olive groves were kept ‘clean’ with very short grass by grazing and by burning the slopes of earthen terraces. In the Monte Pisano three shepherds (two now retired) flatly denied ever having used fire in order to improve pasture, but one told us that ‘at the time of my grandfather’ fire had been used to eliminate brambles and brush from pastures, and that this practice had been stopped by the forest service. This agrees with accounts of two former peasants in the provinces of Siena and Pisa, who told us that shepherds used to burn patches of brush and brambles (*Rubus spp.*), a practice also described for the Apennines (Benatti et al. 2019) and in Liguria (Cevasco 2012). One shepherd told us that he had maintained pastures solely by fertilizing and cutting brush, another that burning ‘was done’ to maintain pastures. These accounts agree with ecologists’ finding that heavy grazing can maintain grasslands for long periods of time (Bond 2019; Stevens et al. 2022). It seems likely that pastures were maintained through a combination of intensive grazing, manual brush cutting and fertilization, and in some places and at some times, by managed burning. In Italy, as in most Mediterranean countries, pastoral fire has been intensely stigmatized, making traditional managed burning poorly understood, although experimental recreations (Cevasco et al. 2020a), and collaborations between prescribed burning and traditional managed burning practitioners have taken place in a few areas (Ascoli and Bovio 2013). While historical ecologists, anthropologists, and historians are certain that fire was a key tool of historic agrosilvopastoral land management, there is little detail on how and when farmers and shepherds used fire. Our research on former burning practices, limited as it is, addresses this absence.

### Qualitative transect walks

In a transect walk from the Vorno to Calci mountains in 2014, Mathews and a botanist assistant recorded qualitative evidence forest species composition and stand structure and recorded a dense scrub and a leaf litter 5–10 cm deep in young maritime pine stands at lower and middle elevations (Mathews 2018, 2022). There was less leaf litter in areas of oak and chestnut coppice at higher elevations. Fallen branches, tree heather, and brambles created potential fire ladders in abandoned chestnut groves.

### Ethnographic study of community response to recent fires

Since the 1970s, the area burned on the Monte Pisano has averaged about 95 hectares per year, with occasional large fires making up the bulk (Tonarelli 2019), and with 80% of the pine forest on lower slopes burning least once in since 1970 (Bertacchi 2022). In repeatedly burned areas, flammable scrub species such as gorse and tree heather regenerate prolifically, making future fires still more likely. The relatively small fires in unmanaged pastures and woodlands in the wake of land abandonment in the 1960s, have given way to occasional large and intense summer fires in abandoned pine forests and scrub. A 2800 hectare fire in 1971 that killed two people led to the organization of volunteer fire brigades in 1976 (Casella 2015), and fire suppression is widely supported by the community. We have carried out ethnographic research on fire management since 2016, interviewing fire managers and attending meetings of fire-fighting organizations.

**Fig. 5:**
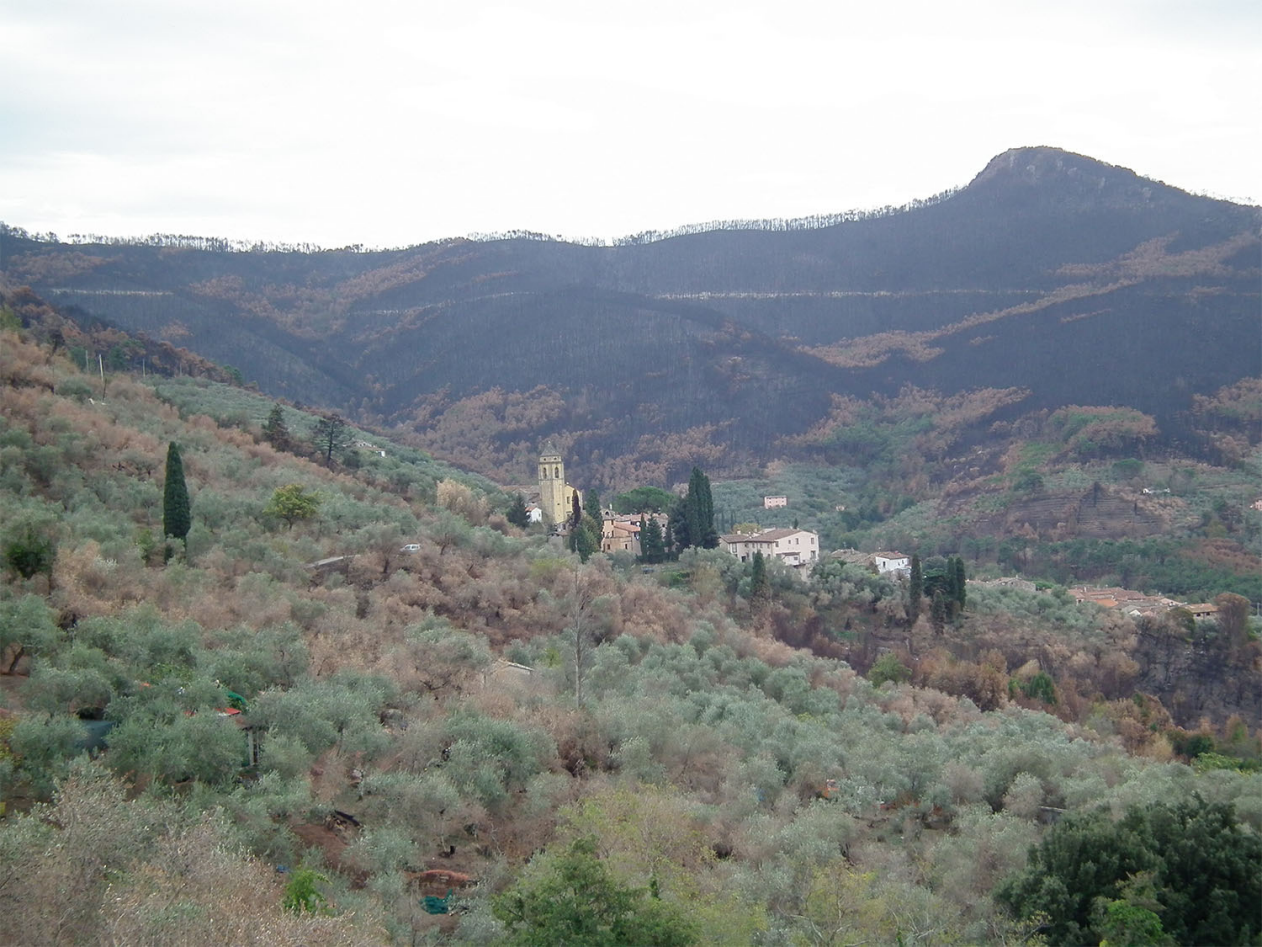
Valley of Calci after fires in September 2018. (Picture by Fabio Malfatti, 2018)

On September 24, 2018, a highly atypical late season wind propelled a fire which grew rapidly to burn 1200 hectares, destroying four homes, damaging seven others (Fig. 5), and threatening the important Certosa monastery in Calci (Gori 2018). Fire fighters came from across Italy, deploying the full apparatus of modern fire suppression, including water dumping airplanes. Post fire analysis found, unsurprisingly, that it was abandoned pine forest and Mediterranean scrub on the lower slopes of the mountains that burned most easily. Olive groves that had been cleared of grass were relatively fire resistant, while overgrown olive groves burned easily and spread fire to nearby houses. In 2019 we interviewed municipal leaders in five towns around the Monte Pisano. Malfatti has carried out participant observation in numerous community meetings where fires and post fire responses were discussed. There was partial agreement about the causes of the fire between our elderly informants and senior fire-fighting officials. Older people attributed wildfires to the abandonment of managed burning, litter raking, grazing, and firewood gathering. Fire managers were deeply worried about the legacies of agropastoral abandonment and some knew of the abandonment of litter raking, but they did not talk about traditional managed burning. They were most concerned that the accumulation of dry vegetation would lead to further fires unless fuel loads were reduced through prescribed burning. Elected officials and local residents agreed that it was the general lack of land care which made the fire so large and dangerous, but most did not know the details of traditional practices which formerly made the landscape less flammable. Media accounts of this fire, as of other wildfires in Italy, celebrated the heroism of firefighters and community members and emphasized fire suppression (Gori 2018). A smaller fire caused by an effort to burn brush in 2019 (Bianchi 2019) was blamed by media and by our informants on the landowner’s ignorance and incompetence. This is a pattern found across Italy, where media account blame wildfires on criminality, lack of care, and on shepherds. This blind spot to traditional grazing and burning practices is a cultural legacy of historic fire regulations and of state attitudes to peasants and pastoralists. Fire managers’ efforts to introduce prescribed burning are rendered more difficult by this legacy. In the Monte Pisano, in contrast, strong communication between community leaders and fire managers, together with a better understanding of land abandonment processes, has inspired a range of proposals to reduce fuel loads, from prescribed grazing to brush clearing, to prescribed burning (Fig. 4).

## Conclusion

Through a combination of oral histories, census data, and contemporary historical and agronomical writing, we have shown that litter raking for fertilizer, carried out by women, was formerly widespread across the Monte Pisano. Litter raking extracted 30–40% of net primary productivity and made a major contribution to reducing the flammability of the landscape. The abandonment of litter raking and firewood gathering in forests, and of managed burning on agricultural terraces and pastures, beginning in the 1960s, has made the landscape of the Monte Pisano much more fire prone than in the past. The contemporary increase in wildfires is the result of a combination of increased fuel loads due to land abandonment and increased summer temperatures and droughts due to climate change. Litter raking was likely much more widespread across Italy and the Mediterranean than has been assumed. Like other traditional practices, litter raking is best studied through a combination of oral history and historical ecological methods. On the Monte Pisano litter raking was largely carried out by women and children. It was likely ignored by officials because was a gendered, nonmodern ‘secondary practice’ outside the market economy. Although the importance of abandoning litter raking in setting the stage for wildfires is known to a few officials and fire managers in the Monte Pisano, the intense stigma around fire means that the role of traditional burning practices in reducing fire risk is much less widely known. Historically agricultural and pastoral fires were ignored by officials because they were illegal but often tolerated.

Our interviews and analysis of media find that the role of traditional agricultural and pastoral practices in reducing wildfires is little known to the general public and to most officials. A cultural legacy of official ignorance is the popular response to present-day fires, which focuses largely on fire suppression. There was little scientific research to support historical state hostility to fire. A legacy of this hostility is a relative lack of scientific studies on traditional burning in the Mediterranean (for Italy see Ascoli and Bovio 2013). Communicating the importance of traditional agropastoral burning, firewood cutting, and litter raking could support current efforts reduce fire risk through prescribed burning and grazing. Efforts to reduce fire risk in the Mediterranean and around the world would benefit from ethnographic research on agropastoral practices that have been ignored due to official stigma (such as burning) or which are carried out by socially marginalized groups (such as litter raking by women). The rapport produced through long-term ethnographic research is critical to investigating the mundane practices of hitherto ignored people. Ethnohistorical and historical ecology research on traditional agropastoral practices can help us understand their cultural and ecological legacies in contemporary landscapes. Our Mediterranean case is therefore applicable to world regions where land abandonment is causing reductions in burned area, as in Africa and parts of Latin America (Jones et al. 2022).

Our finding that litter raking was widespread in Italy suggests that its effects may be underappreciated by ecologists in other parts of the world. Litter raking is or was an important practice as far afield as Nepal (Giri and Katzensteiner 2010), Japan (Hiratsuka et al. 2020), and Korea (Hong et al. 1995). It is often women’s work: pictures of litter raking in early twentieth Switzerland show women (Bürgi et al. 2013), conflicts over litter raking in nineteenth-century Moravia involved women (Szabò 2021), studies of litter raking in south China show women (Brown et al. 1995), and litter raking is done by women in contemporary Turkey (Ekin Kurtic pers. com 2023). The division of labor which makes litter raking gendered work may be a recent phenomenon, but it may have a deeper history in many places. There is a strong likelihood that, as in Italy, its ecological effects may be overlooked by scientists and officials because of the gender and socioeconomic status of people who rake litter. It is likely that in many places women’s work formerly made landscapes less vulnerable to fire. In other places it continues do so.

## Footnotes

1 In this paper we use ‘managed burning’ to refer to traditional agropastoral burning and ‘prescribed burning’ to refer to modern burning practices introduced to southern Europe since the 1970s (Ascoli and Bovio 2013).

2 We have used pseudonyms for all of our informants except Fabio Casella and Fausto Giorgi.
